# Exposure Data of Cosmetic Products for Patients Undergoing Cancer Treatment

**DOI:** 10.1111/jocd.70438

**Published:** 2025-10-13

**Authors:** Pauline Roch‐Simon, Audrey Bernard, Sandrine Rakotomalala, Marie‐Pierre Gomez‐Berrada, Pierre‐Jacques Ferret

**Affiliations:** ^1^ Pierre Fabre Dermo‐Cosmétique Toulouse France

**Keywords:** cancer treatments, consumption habits, dermo‐cosmetic products, exposure data, frequency data, quantity data

## Abstract

**Background:**

Dermo‐cosmetics play a major role in the management of cutaneous side effects due to cancer treatments. Before marketing cosmetic products, a safety assessment using realistic exposure data must be performed. However, limited data are available for patients undergoing cancer treatments.

**Aims:**

To obtain information about consumption habits and cosmetic product quantities applied by this specific population.

**Methods:**

196 participants, between 31 and 86 years old, who have had cancer, were recruited to compose a skincare (*N* = 150) and a makeup panel (*N* = 46). Participants completed a questionnaire inquiring about their cancer, treatments, and associated side effects. Frequencies of use of 31 cosmetic and 11 makeup products during and after treatment and changes in consumer behavior were collected. Quantities of five skincare and 10 makeup products were obtained, respectively, through a one‐week consumption follow‐up and a makeup workshop.

**Results:**

88% of the panel have had breast cancer and 98% reported at least one cutaneous side effect due to their oncological treatment. More than 80% of the participants have modified their cosmetic consumption since the beginning of their treatment. For 22 products, statistically significant differences between frequency of use during treatment and post‐treatment were highlighted. Differences between quantities from this study and already published data were emphasized. For example, participants applied a higher quantity of body balm than the general population.

**Conclusion:**

This study provides information on cosmetic consumption habits and quantity data for patients undergoing cancer treatment. These results will allow a more specific exposure and safety assessment for this population.

## 1 Introduction

According to data published by the International Agency for Research on Cancer (IARC), in 2020, there were 19.3 million of new cancer cases, worldwide [1]. It is estimated that one in five people will suffer from cancer during their lifetime [2]. Different treatments can be prescribed to patients in order to optimize their cancer therapeutic management. Nevertheless, these treatments can cause different side effects on skin, hair and nails, such as xerosis, hand foot syndrome, alopecia and nail disorders [3]. They affect the patient's quality of life, their self‐image and sometimes even impact patient compliance with treatment. As mentioned in a 2015 paper by Kambil et al., “Toxic effects on skin, hair and nails can negatively affect the quality of life and also lead to interruption or discontinuation of these drugs” [4].

Dermo‐cosmetics play a major role in the management of these cutaneous side effects. Indeed, dermo‐cosmetic products can minimize skin xerosis' intensity by increasing skin hydration, which can significantly improve the patient's quality of life [5]. However, Article 3 of the European Regulation on cosmetic products N°1223/2009 [6] requires every marketed cosmetic product to be safe for consumers. Thus, having realistic exposure data for the specific target populations is essential to conduct an accurate safety assessment. Unfortunately, available data concerning patients undergoing cancer treatment is limited. Due to their health condition, these subjects' specific population may not have the same cosmetic product consumption as the general population. They could adapt their usage pattern; for instance, use a product more or less frequently or with a higher or lower quantity. Consequently, this study aims to obtain reliable information regarding consumption habits and exposure data (such as frequencies of use and quantities of cosmetic products used) for patients undergoing cancer treatment.

## 2 Materials and Methods

### 2.1 Skincare Product Survey

#### 2.1.1 Study Population

150 participants were recruited among patients sojourning in the Avène thermal station, following a cancer therapy. Participants were included from June 2021 to October 2021, after a medical agreement and signature of a consent form. The inclusion criteria were:

- Being an adult (over 18 years old) male or female in thermal cure;
- Undergoing or having undergone oncological treatment.

No oncological treatment was administered during the stay at the station, except for the hormonal therapy.

All participants were French citizens coming from 51 different departments.

#### 2.1.2 Study Design

The study was divided into two parts: a questionnaire and a consumption follow‐up.

##### 2.1.2.1 Questionnaire

In order to improve knowledge on consumption habits of patients undergoing cancer treatment, a survey has been designed. At the beginning of the questionnaire, a medical professional completed the body weight and the phototype of each patient. The other questions had to be filled out by panelists before the end of their stay at the station.

The questionnaire enquired about the respondent's type of cancer, oncological treatments and their associated side effects. Subjects were also interviewed about their cosmetic consumption habits. Frequencies of use during oncological treatment and post‐treatment were collected for 31 cosmetic products. Those products were divided into several categories: cleanser products, moisturizing products, sunscreen products, makeup products, complementary products and oral care products. Three different scales were defined to collect the frequencies of use according to Table 1. As some frequencies of use in the questionnaire were presented as a range, a single value has been assigned using the following method: “2 to 4 times a week” corresponding to “3 times a week”, “more than twice a day” corresponding to “3 times a day” and “more than 4 times a day” corresponding to “5 times a day”. Subjects were also asked about their cosmetic product selection criteria, changes in consumer behavior since the beginning of their oncological treatment and places of purchase of cosmetic products. Demographic data such as sex, age, marital status, place of residence and socio‐professional category were also collected.

**TABLE 1:** Type of products and their corresponding scale.

Type of product	*N*	Frequency scale		
		Scale 1	Scale 2	Scale 3
Cleansers	6	Shower gel, cleansing oil, face cleanser, makeup remover, shampoo	Soap	
Moisturizers	7	Face cream, hydrating face mask, eye contour, body moisturizer, foot cream, lip balm, hand cream		
Sunscreen products	2		Sunscreen cream/milk/spray, sunscreen stick	
Makeup products	8			Concealer, liquid foundation, powder foundation, eyebrow pencil, mascara, eye pencil, lipstick, fortifying nail polish
Complementary products	5	Deodorant, soothing scalp lotion, hair mask	Thermal water spray, repairing cream	
Oral care products	3		Toothpaste, mouthwash, oral gel	

*Note:* Scale 1: Never, once a week, two to four times a week, once a day and twice a day. Scale 2: Never, once a week, two to four times a week, once a day, twice a day, three times a day, four times a day and more than four times a day. Scale 3: Never, once a week, two to four times a week, once a day, twice a day and more than twice a day.

##### 2.1.2.2 Consumption Follow‐Up

Among the 150 participants who completed the survey, 149 agreed to participate in a consumption follow‐up. Five commercialized products assessed for the population undergoing cancer therapy and having good cosmetovigilance records were provided to the participants: a body cleansing oil, a face moisturizer, a body moisturizer balm, a hand cream, and a face and body sunscreen cream.

During the consumption follow‐up, the subjects recorded daily usage information over a one‐week period. On the one hand, if they were already using a product, the participants were asked to use the product as a replacement for their usual product, without changing their routine. On the other hand, if they were not, they were advised to use the product in accordance with its instructions of use. In order not to bias the results, participants were reminded that the products were for personal use only and could not be lent to anybody.

In order to determine the total amount of product used, products were weighed by the staff using a precision scale with a sensitivity of 0.01 g, at the beginning and at the completion of the consumption follow‐up. Frequency of use, quantity of use per day and per application were determined from the consumption follow‐up data for each of the five investigated products.

#### 2.1.3 Data Analysis

##### 2.1.3.1 Questionnaire

Descriptive statistics were performed using Excel for demographic, cancer‐related (type of cancer, oncological treatment and side effects), usage frequency, product selection criteria and consumption modification data. Statistical tests between data before and after oncological treatment were carried out using StatTools software (version 8.2). Paire‐wise comparison tests (Wilcoxon rank‐sign test) were performed on frequency of use and on product selection criteria in order to determine whether there is a statistically significant difference at a 5% level between data from the two studied periods: during treatment and post‐treatment.

##### 2.1.3.2 Consumption Follow‐Up

The panelists' exposure to the five investigated cosmetic products was assessed using a probabilistic method. Non‐user data and outlier quantity data such as a negative amount of product applied, were eliminated in order not to distort the exposure calculation defined by the following equation:

$$\text{Exposure}\;\left(\mathrm{mg}/\mathrm{kg}\;\mathrm{bw}/\mathrm{day}\right)=\frac{F\times Q\times 1000\times \mathrm{RF}}{\mathrm{BW}}$$

BW, Body weight (kg); F, Frequency of use (day^−1^); Q, Quantity per use (g/use); RF, Retention factor.

Frequency, quantity per use and body weight data were adjusted to theoretical distribution using @Risk software (version 8.2). Retention factors were determined according to the recommendations of the Scientific Committee on Consumer Safety (SCCS): 100% for leave‐on product (face cream, body balm, hand cream and sunscreen cream) and 1% for rinse‐off products (cleansing oil) [7].

According to the recommendations of the United States Environmental Protection Agency (US EPA), probabilistic exposure calculation was performed using Monte Carlo random simulations and 10 000 iterations [8].

### 2.2 Makeup Product Survey

#### 2.2.1 Study Population

Forty‐six participants following a cancer therapy were recruited from September 2022 to November 2022. To be included in the study, subjects had to undergo or have undergone an oncological treatment.

All respondents were French citizens coming from 28 departments.

#### 2.2.2 Study Design

Participants of the study completed a questionnaire about their cancer and their oncological treatment with the associated side effects. Their makeup consumption habits were collected to obtain frequencies of use during treatment and post‐treatment for 11 makeup products: a tinted cream, a sunscreen tinted cream, a foundation, a concealer, a translucent powder, a bronzing powder, an eyebrow pencil, an eye pencil, an eye shadow, a mascara and a lipstick. The following frequency scale was used to collect frequencies of use: “Never”, “Once a week”, “2 to 4 times a week”, “Once a day”, “Twice a day” and “More than twice a day”. As some frequencies were ranges, a single value was determined as follows: “2 to 4 times a week” corresponding to “3 times a week” and “More than twice a day” corresponding to “3 times a day”. They were also asked about their consumption change in makeup products since the beginning of their oncological treatment and after the end of their treatment.

The panel took part in a makeup workshop organized at the thermal station in order to obtain frequency of use and quantity applied per application for 10 marketed makeup products: tinted cream, sunscreen tinted cream, foundation, concealer, bronzing powder, translucent powder, eyebrow pencil, eye pencil, mascara and colored lipstick. Participants were asked to use the products as they usually do. These products were weighed before and after application, using a precision scale with a sensitivity of 0.01 mg.

#### 2.2.3 Data Analysis

##### 2.2.3.1 Questionnaire

Descriptive statistics were carried out using Excel on demographic, cancer‐related (type of cancer and end of treatment), skin type and skin sensitivity (during treatment and after treatment), side effects‐related, usage frequency, and consumption modification data. Using StatTools software (version 8.2), a Wilcoxon rank‐sign test was performed to define whether there was a statistically significant difference at a 5% level between usage frequency data for the 11 makeup products from the two investigated periods: during the oncological treatment and post‐treatment.

##### 2.2.3.2 Exposure Data

The exposure assessment to the investigated products was performed as described in Section 2.1.3.2 of the present study.

## 3 Results

### 3.1 Skincare Product Survey

#### 3.1.1 Description of the Study Population

The characteristics of the study population are presented in Table 2.

**TABLE 2:** Characteristics of the studied population.

Characteristics	*N* (Total = 149)	Percentage of participants
**Gender**		
Women	142	95.30%
Men	3	2.01%
Answer not communicated	4	2.68%
**Age (years old)**		
< 40	6	4.03%
40–59	86	57.72%
≥ 60	54	36.24%
Answer not communicated	3	2.01%
**Type of cancer**		
Breast cancers	133	89.26%
Gastrointestinal cancers	3	2.01%
Lymphoma	3	2.01%
Other type of cancers (leukemia, melanoma, leiomyosarcoma, lung cancer, …)	5	3.36%
Answer not communicated	5	3.36%
**Oncological treatment**		
Surgery	138	92.62%
Chemotherapy	101	67.79%
Radiotherapy	130	87.25%
Targeted therapy	13	8.72%
Immunotherapy	9	6.04%
Hormonal therapy	96	64.43%
**Side effects**		
At least one side effect reported	144	96.64%
No side effect reported	4	2.68%
Answer not communicated	1	0.67%
Type of side effect		
Skin dryness	114	76.51%
Sensation of discomfort	96	64.43%
Skin redness	50	33.56%
Desquamation	39	26.17%
Allergy	37	24.83%
Skin rashes	47	31.54%
Hand foot syndrome	92	61.74%
Alopecia	96	64.43%
Conjunctivitis	27	18.12%
Eye oedema	17	11.41%
Lip inflammation	12	8.05%
Lip dryness	40	26.85%
Gingivitis	40	26.85%
Mouth ulcers	47	31.54%
Dry mouth	69	46.31%
Tongue infection	10	6.71%
Mycosis/vulvar itching	33	22.15%
Burning sensation on the vulva/vulvar dryness	48	32.21%
Other (ocular dryness, nail damage, …)	31	20.95%

Among the participants aged from 31 to 86 years old, 95% were women (*N* = 142), 2% were men (*N* = 3) and 3% of the participants (*N* = 4) did not communicate their gender. 89.26% (*N* = 133) of the panel were treated for breast cancer, and 7.38% (*N* = 11) of the participants had other types of cancer, such as lymphoma or colorectal cancer. In order to treat those cancers, more than 76% of the panel received from three to four different oncological treatments. The main therapies received by the participants of the study were surgery (92.62%), radiotherapy (87.25%), chemotherapy (67.79%), and hormonal therapy (64.43%). Those treatments caused at least one side effect among 96% of the participants, and most of them reported at least six side effects. The main side effects were skin dryness (76.51%), the sensation of discomfort on skin (64.43%), alopecia (64.43%) and hand foot syndrome (61.74%) (Table 2). Four participants did not report any side effects. Among them, only one participant received chemotherapy; the others had surgery and received radiotherapy and/or hormonal therapy.

#### 3.1.2 Modifications of Consumption Habits

Since the beginning of the oncological treatment, 82.55% of the participants have modified their consumption. All types and reasons for modification are reported in Table 3. The main types of modification were the adoption of a new routine in accordance with the oncological treatment received (69.11%), for example, using moisturizing products after the radiotherapy and a change in cosmetic product selection criteria (62.60%). The most important criteria were the security of the product (criteria mentioned by 62.42% of the panel during treatment vs. 61.07% post‐treatment), the brand (55.03% during treatment vs. 59.06% post‐treatment), the texture of the product (49.66% during treatment vs. 60.40% post‐treatment) and the presence of natural ingredients in the formula (56.38% during treatment vs. 65.77% post‐treatment). Moreover, some criteria appeared to present a different emphasis according to the studied period. In fact, statistically significant differences at a 5% level were shown for a few product selection criteria. For instance, the price of the product was less important during treatment than post‐treatment (criteria selected by 26.85% of respondents during treatment vs. 45.64% post‐treatment). In contrast, association or influencer advice was more important during treatment than post‐treatment: criteria selected by 27.52% of the panel during treatment versus 13.42% post‐treatment (Table 3).

**TABLE 3:** Modification in cosmetic consumption since the beginning of the treatments and product selection criteria during treatment and post‐treatment.

	*N* (Total = 149)	Percentage of participants
**Type of modification in the consumption of cosmetic products**		
Reduction in the number of products	41	33.33%
Increase in the number of products	49	39.84%
Reduction in the quantity of product	11	8.94%
Increase in the quantity of product	40	32.52%
Change in product format	13	10.57%
Change of the place of purchase	39	31.71%
Adoption of a new routine	85	69.11%
Change in product selection criteria	77	62.60%
**Reasons for modification in the consumption of cosmetic products**		
Health professional advice	89	72.36%
Incompatibility feeling	23	18.70%
New needs	90	73.17%
Caution	47	38.21%
Take time for yourself	73	59.35%
**Product selection criteria: during treatment**		
Price*	40	26.85%
Security	93	62.42%
Brand	82	55.03%
Scent, fragrance	54	36.24%
Texture*	74	49.66%
Packaging	3	2.01%
Organic product	59	39.60%
Natural ingredient*	84	56.38%
Advertising	3	2.01%
Association or influencer advice*	41	27.52%
Product scanning application*	10	6.71%
Other	14	9.40%
**Product selection criteria: post‐treatment**		
Price*	68	45.64%
Security	91	61.07%
Brand	88	59.06%
Scent, fragrance	58	38.93%
Texture*	90	60.40%
Packaging	7	4.70%
Organic product	67	44.97%
Natural ingredient*	98	65.77%
Advertising	6	4.03%
Association or influencer advice*	20	13.42%
Product scanning application*	18	12.08%
Other	10	6.71%

*Note:* *p < 0.05.

A variety of reasons for those modifications in cosmetic consumption were given by the participants. The major reasons were the appearance of new needs in terms of cosmetic products (73.17%), advice given by health professionals (72.36%) and the need to take time for themselves (59.35%).

#### 3.1.3 Frequencies of Use

The frequencies of use collected during the study are presented in Table 4.

**TABLE 4:** Percentage of users and frequency of use of 31 cosmetic products during treatment and post‐treatment.

	During oncological treatment					Post oncological treatment				
	Users (%)	Frequency of use (day^−1^)				Users (%)	Frequency of use (day^−1^)			
		Mean	Median	SD	P90		Mean	Median	SD	P90
**Cleansing products**										
Shower gel	71.80	1.06	1.00	0.43	2.00	73.20	1.07	1.00	0.47	2.00
Cleansing oil	47.00	0.97	1.00	0.50	2.00	47.70	0.94	1.00	0.51	2.00
Face cleanser	41.60	1.08	1.00	0.55	2.00	42.30	1.04	1.00	0.58	2.00
Makeup remover*	53.70	1.13	1.00	0.61	2.00	55.70	1.15	1.00	0.58	2.00
Shampoo*	79.90	0.44	0.43	0.27	1.00	94.60	0.44	0.43	0.27	1.00
Soap	43.60	1.99	1.00	1.47	4.00	41.60	2.19	2.00	1.59	5.00
**Moisturizing products**										
Face cream	95.97	1.56	2.00	0.55	2.00	94.63	1.57	2.00	0.52	2.00
Face mask	37.58	0.22	0.14	0.21	0.43	38.93	0.17	0.14	0.13	0.14
Eye contour	39.60	0.94	1.00	0.61	2.00	36.91	0.84	1.00	0.60	2.00
Body moisturizer*	94.63	1.22	1.00	0.67	2.00	94.63	0.97	1.00	0.61	2.00
Foot cream*	70.47	0.79	1.00	0.59	2.00	68.46	0.59	0.43	0.49	1.00
Lip balm*	73.15	1.25	1.00	0.73	2.00	66.44	1.03	1.00	0.74	2.00
Hand cream*	82.55	1.22	1.00	0.69	2.00	83.89	0.98	1.00	0.66	2.00
**Sunscreen products**										
Sunscreen cream/milk/spray	71.14	1.83	1.50	1.38	4.00	82.55	1.40	1.00	1.21	3.00
Sunscreen stick	18.12	2.15	2.00	1.66	5.00	17.45	1.68	1.00	1.43	3.50
**Makeup products**										
Concealer	27.52	0.74	1.00	0.56	1.00	32.21	0.63	0.43	0.42	1.00
Liquid/cream foundation	36.24	0.74	1.00	0.37	1.00	44.97	0.65	1.00	0.39	1.00
Compact powder foundation	25.50	0.68	0.43	0.47	1.00	32.89	0.61	0.43	0.41	1.00
Eyebrow pencil*	44.97	1.03	1.00	0.65	2.00	38.26	0.87	1.00	0.61	2.00
Mascara*	30.20	0.65	0.43	0.41	1.00	63.09	0.62	0.43	0.40	1.00
Eye pencil*	37.58	0.86	1.00	0.61	2.00	55.70	0.68	0.43	0.46	1.00
Lipstick*	42.95	0.81	0.43	0.79	2.00	57.72	0.75	0.43	0.79	2.00
Fortifying nail polish*	72.48	0.44	0.43	0.49	1.00	60.40	0.24	0.14	0.20	0.43
**Additional products**										
Thermal spring water spray*	66.44	1.70	2.00	1.35	3.00	59.06	1.12	1.00	0.96	2.00
Repairing cream*	84.56	1.70	1.00	1.20	3.00	63.76	1.03	1.00	0.84	2.00
Deodorant*	40.96	1.04	1.00	0.46	2.00	65.77	1.07	1.00	0.41	2.00
Soothing scalp lotion*	32.21	0.93	1.00	0.73	2.00	18.12	0.44	0.14	0.59	1.40
Hair mask/conditioner*	36.91	0.35	0.43	0.19	0.43	56.38	0.31	0.43	0.18	0.43
**Oral care products**										
Toothpaste*	98.66	2.48	2.00	0.70	3.00	97.99	2.32	2.00	0.66	3.00
Mouthwash*	72.48	1.80	2.00	1.47	4.00	39.60	0.66	0.43	0.84	1.20
Oral gel*	13.42	1.44	1.00	1.45	3.10	6.04	1.10	0.43	1.12	3.00

*Note:* *p < 0.05.

Variations in frequencies of use between the two investigated periods (during oncological treatment and post‐treatment) have been observed for the 31 cosmetic products and with statistically significant differences at a 5% level for 19 of them.

The body moisturizer was applied more frequently during treatment than post‐treatment (mean frequency: 1.22 day^−1^ vs. 0.97 day^−1^) but the percentage of users was identical during both periods (94.63% of users). Concerning foot cream and lip balm, the mean frequency of use and the percentage of users during treatment were higher than post‐treatment (foot cream: 0.79 day^−1^ and 70.47% vs. 0.59 day^−1^ and 68.46%; lip balm: 1.25 day^−1^ and 73.15% vs. 1.03 day^−1^ and 66.44%). Although the percentage of hand cream users was similar between both periods (82.55% vs. 83.89%), the product was applied more frequently during treatment (mean: 1.22 day^−1^ vs. 0.98 day^−1^). In contrast, the shampoo was used at the same frequency during both periods, but the percentage of users was lower during treatment (79.90% vs. 94.60%).

Percentage of users of fortifying nail polish was higher during treatment than post‐treatment (72.48% vs. 60.40%) and users had increased their frequency of use during oncological treatment (mean frequency of 0.44 times a day vs. 0.24 times a day). Percentage of users of soothing scalp lotion and mouthwash was higher during treatment than post‐treatment: 32.21% versus 18.12% for the soothing scalp lotion and 72.48% versus 39.60% for the mouthwash. Participants also increased their frequency of use during treatment for these two products: 0.93 times a day versus 0.44 times a day for the soothing scalp lotion and 1.80 times a day versus 0.66 times a day for the mouthwash (mean values).

#### 3.1.4 Exposure Data

Exposure assessment to five cosmetic products often used by patients undergoing cancer treatment has been performed thanks to data generated by a consumption follow‐up. Frequencies of use, quantities per application and per day, and exposure are presented in Table 5.

**TABLE 5:** Percentage of users and exposure data for the five investigated products.

Product categories	Users (%) *N* = 149	Frequency of use (day^−1^)				Quantity per application (g/use)				Quantity per day (g/day)				Exposure (mg/kg bw/day)			
		Mean	Median	SD	P90	Mean	Median	SD	P90	Mean	Median	SD	P90	Mean	Median	SD	P90
Cleansing oil	99.33	1.30	1.19	0.56	2.03	5.20	4.03	4.18	10.00	6.71	4.78	6.47	13.70	1.04	0.73	1.08	2.12
Face cream	99.33	1.82	1.71	0.68	2.70	0.43	0.39	0.20	0.69	0.79	0.67	0.49	1.37	12.06	9.94	8.03	22.18
Body balm	100.00	1.34	1.19	0.67	2.19	5.27	4.58	3.01	9.05	7.07	5.51	5.80	13.74	107.98	83.31	90.89	212.02
Hand cream	91.95	1.49	1.18	1.18	2.85	0.69	0.57	0.48	1.26	1.02	0.67	1.16	2.18	15.62	10.07	19.40	33.30
Sunscreen cream	83.89	0.63	0.45	0.57	1.30	2.16	1.46	2.34	4.53	1.36	0.67	2.22	3.17	20.68	9.95	35.33	47.22

The cleansing oil was used 2.03 times a day by the panel with a quantity of 10.00 g/use (P90 values). Thus, the exposure value at the 90th percentile was 2.12 mg/kg bw/day.

At the 90th percentile, participants used 0.69 g of face cream per application with a frequency of use of 2.70 times a day and an exposure value of 22.18 mg/kg bw/day.

The panel used the body balm with a frequency of 2.19 times a day with a quantity of 9.05 g per use (P90 values). Therefore, at the 90th percentile, the exposure was 212.02 mg/kg bw/day.

The exposure value at the 90th percentile for hand cream was 33.30 mg/kg bw/day, with a frequency of use of 2.85 times a day and a quantity of 1.26 g per application.

The sunscreen cream was used by the panel 1.30 times a day with a quantity of 4.53 g per application (P90 values). The 90th percentile of exposure calculated was 47.22 mg/kg bw/day.

### 3.2 Makeup Product Survey

#### 3.2.1 Description of the Study Population

All the data stated below are presented in Table 6.

**TABLE 6:** Characteristics of the studied population.

Characteristics		*N* (Total = 46)	Percentage of participants
Gender	Women	46	100%
Age (years old)	< 40	2	4.35%
	40–59	26	56.52%
	≥ 60	18	39.13%
Skin type on the face: during treatment	Normal skin	3	5.52%
	Dry skin	36	78.26%
	Combination skin	5	10.87%
	Answer not communicated	2	4.35%
Skin type on the face: post‐treatment	Normal skin	7	15.22%
	Dry skin	23	50.00%
	Combination skin	13	28.26%
	Answer not communicated	3	6.52%
Skin sensitivity/reactivity on the face: during treatment	Normal skin	4	8.70%
	Sensitive skin	18	39.13%
	Reactive skin	22	47.83%
	Answer not communicated	2	4.35%
Skin sensitivity/reactivity on the face: post‐treatment	Normal skin	14	30.43%
	Sensitive skin	22	47.83%
	Reactive skin	8	17.39%
	Answer not communicated	2	4.35%
Type of cancer	Breast cancers	39	84.78%
	Other type of cancers (lung, gastrointestinal, …)	7	15.22%
Treatment progress^a^	Treatment completed	42	91.30%
	Treatment still ongoing	4	8.70%
Duration since the end of treatment^a^	Less than 3 months	6	14.29%
	Between 3 and 6 months	11	26.19%
	Between 6 months and a year	5	11.90%
	More than a year	20	47.62%
Hormonal therapy administration	Patient has received hormonal therapy	25	54.35%
	Patient has not received hormonal therapy	20	43.48%
	Answer not communicated	1	2.17%
Duration since the beginning of the hormonal therapy	< 3 months	4	16.00%
	Between 3 and 6 months	3	12.00%
	Between 6 months and a year	2	8.00%
	Between a year and 5 years	12	48.00%
	Between 5 and 10 years	4	16.00%
Total duration of the hormonal therapy	Between 5 and 10 years	23	92.00%
	More than 10 years	2	8.00%
Type of side effects on the face	Skin dryness	36	78.26%
	Sensation of discomfort	20	43.48%
	Redness	17	36.96%
	Rash	10	21.74%
	Eyebrow loss	35	76.09%
	Eyelash loss	31	67.39%
	Lip dryness	32	69.57%
Number of reported side effects per participants	0	0	0%
	1	2	4.35%
	2	10	21.74%
	3	6	13.04%
	4	14	30.43%
	5	4	8.70%
	6	5	10.87%
	7	5	10.87%

^a^ Excluding hormonal therapy.

All study participants were women aged from 31 to 77 years old, with the mean age being 56 years old. 84.78% of the panel had breast cancer, and 15.22% were suffering from another type of cancer, for instance, lung, skin or colorectal cancer. Excluding hormonal therapy, oncological treatments were achieved for 91.30% of the participants. Among them, 14.29% have completed their treatment since < 3 months, 26.19% between 3 and 6 months, 11.90% between 6 months and a year, and 47.62% for more than a year. 54.35% of respondents had hormonal therapy, but they were at different stages in their treatment. 16.00% of them were undergoing hormonal therapy for < 3 months, 12.00% from 3 to 6 months, 8.00% from 6 months to a year, 48.00% from a year to 5 years, and 16.00% from 5 years to 10 years. The total duration of the hormonal therapy was between 5 and 10 years for 92.00% of the panel and more than 10 years for 8.00% of the study participants.

The subjects had different facial skin types and skin sensitivity/reactivity on their face between the period during treatment and the period post‐treatment. During the oncological treatment, there were more participants with dry skin than post‐treatment (respectively, 78.26% vs. 50.00%). On the other hand, throughout the post‐treatment period, more participants had a normal skin type and combination skin than during cancer treatment (normal skin type: 5.52% vs. 15.22% and combination skin type: 10.87% vs. 28.26%). More respondents declared having skin sensitivity on the face after their oncological treatment (39.13% during treatment vs. 47.83% post‐treatment). 47.83% of the panel reported skin reactivity on their face during treatment (vs. 17.39% post‐treatment).

All respondents declared having side effects on the face due to cancer treatment. The main side effects observed were skin dryness (78.26%), eyebrow loss (76.09%), lip dryness (69.57%) and eyelash loss (67.39%). The mean number of side effects reported by the participants was 4.

#### 3.2.2 Makeup Routine Modifications

As described in Table 7, 67.39% of the participants have stopped or decreased their makeup consumption during their oncological treatment. Respondents have selected different reasons: 61.29% of the panel were too tired to apply makeup and 58.06% did not want to wear makeup. In contrast, 36.96% of study participants started a makeup routine during their treatment. The main mentioned reasons were to have healthy glowing skin (76.47%) and to redraw their eyebrows (76.47%).

**TABLE 7:** Makeup routine modification since the beginning of the oncological treatment.

		*N* (Total = 46)	Percentage of participants
**Stop or decrease in makeup consumption during oncological treatment**			
Stop or decrease		31	67.39%
No modification		15	32.61%
Reasons for modification (*N* = 31)	Difficulties to apply makeup	9	29.03%
	Does not want to	18	58.06%
	Too tired	19	61.29%
	Price	3	9.68%
	Other	10	32.26%
**Start of a makeup routine since the beginning of treatment**			
Start of a makeup routine		17	36.96%
No modification		27	58.70
No answer communicated		2	4.35%
Reasons to start a makeup routine during oncological treatment (*N* = 17)	To have healthy glow	13	76.47%
	To redraw eyebrows	13	76.47%
	To emphasize their eyes	10	58.82%
	To apply makeup on their lips	5	29.41%
	Hide a scar	1	5.88%
	Other	1	5.88%
**Modifications of makeup routine since the beginning of treatment**			
Modification		37	80.43%
No modification		8	17.39%
No answer communicated		1	2.17%
Type of makeup routine modifications (*N* = 37)	Decrease in the number of products used	13	35.14%
	Increase in the number of products used	8	21.62%
	Decrease in the quantity of product applied	3	8.11%
	Increase in the quantity of product applied	5	13.51%
	Modification in the type of products	26	70.27%
	Change of brand	23	62.16%
	Other	5	13.51%
Reasons for makeup routine modifications (*N* = 37)	New needs	18	48.65%
	Feeling of incompatibility	14	37.84%
	Take care of themselves	19	51.35%
	Other	7	18.92%

80.43% of the subjects declared having modified their makeup routine since the beginning of their cancer treatment. The main types of modifications were a change in the type of products used for 70.27% of the panel and a modification of makeup brand for 62.16% of the respondents. They modified their makeup routine because of various reasons. 51.35% of the participants modified their makeup consumption in order to take care of themselves, and 48.65% mentioned the emergence of new needs in terms of makeup products since the beginning of their oncological treatment.

#### 3.2.3 Frequencies of Use

Frequencies of use of 11 investigated products are presented in Table 8.

**TABLE 8:** Frequency of use of 11 makeup products during oncological treatment and post‐treatment.

Product categories	Frequency of use during treatment (day^−1^)				Frequency of use post‐treatment (day^−1^)			
	Mean	Median	SD	P90	Mean	Median	SD	P90
Tinted cream	0.31	0.00	0.52	1.00	0.44	0.14	0.50	1.00
Tinted sunscreen cream	0.30	0.00	0.67	1.00	0.26	0.00	0.58	1.00
Foundation	0.17	0.00	0.30	0.43	0.27	0.07	0.39	1.00
Concealer*	0.26	0.00	0.55	1.00	0.37	0.07	0.57	1.00
Translucent powder*	0.29	0.00	0.48	1.00	0.38	0.00	0.57	1.00
Bronzing powder*	0.49	0.43	0.44	1.00	0.68	0.72	0.53	1.00
Eyebrow pencil*	0.82	1.00	0.89	2.00	0.51	0.14	0.72	1.50
Eye pencil*	0.43	0.14	0.59	1.00	0.72	0.43	0.76	1.00
Eye shadow*	0.32	0.14	0.41	1.00	0.52	0.43	0.50	1.00
Mascara*	0.42	0.14	0.54	1.00	0.74	1.00	0.59	1.00
Lipstick	1.16	1.00	1.14	3.00	1.34	1.00	1.05	3.00

*Note:* *p < 0.05.

Statistically significant differences have been highlighted for seven makeup products: the concealer, the translucent powder, the bronzing powder, the eyebrow pencil, the eye pencil, the eye shadow, and the mascara. Study participants applied the mascara with a lower frequency during their oncological treatment: the mean frequency was 0.42 times a day during treatment and 0.74 times a day post‐treatment. The eye pencil and the eye shadow were also used by participants with a lower frequency during treatment (eye pencil: 0.43 times a day during treatment versus 0.72 times a day post‐treatment and eye shadow: 0.32 times a day vs. 0.52 times a day post‐treatment). In contrast, they used the eyebrow pencil with a higher frequency during their cancer treatment: 0.82 times a day versus 0.51 times a day.

#### 3.2.4 Exposure Data

None of the makeup workshop's participants used the tinted sunscreen cream, thus the exposure assessment to this product could not be carried out.

Percentage of users, frequency of use, quantity per application, and exposure for nine investigated products are presented in Table 9.

**TABLE 9:** Percentage of users and exposure data for nine makeup products.

Product categories	Users (%)	Frequency of use (day^−1^)			Quantity per application (mg/use)			Exposure (mg/kg bw/day)		
		Mean	SD	P90	Mean	SD	P90	Mean	SD	P90
Tinted cream	34.78	0.87	0.41	1.00	351.70	129.70	521.50	4.56	3.12	8.32
Foundation	34.78	0.54	0.38	1.01	172.60	118.70	314.80	1.44	1.61	3.38
Concealer	36.96	0.65	0.39	1.02	16.51	13.39	31.93	0.15	0.18	0.35
Translucent powder	39.13	0.67	0.47	1.03	44.91	36.56	94.48	0.44	0.57	1.07
Bronzing powder	78.26	0.81	0.49	1.04	32.40	27.23	69.14	0.42	0.51	1.02
Eyebrow pencil	45.65	0.99	0.76	2.00	1.48	0.57	2.22	0.02	0.02	0.05
Eye pencil	41.30	0.80	0.63	1.00	1.89	1.59	3.70	0.02	0.03	0.06
Mascara	89.13	0.80	0.57	1.00	16.70	11.24	30.30	0.21	0.23	0.46
Lipstick	89.13	1.43	0.99	3.00	8.04	10.57	15.15	0.19	0.36	0.41

The mascara, the lipstick, and the bronzing powder were the most used products during the makeup workshop (mascara and lipstick: 89.13% of users; bronzing powder: 78.26% of users). Less than half of the panel applied the tinted cream, the foundation, the concealer, the translucent powder, the eyebrow pencil, and the eye pencil during the workshop.

The foundation was the least frequently used product (0.54 times a day) whereas the lipstick was the most frequently used product (1.43 times a day) by the panelists.

On the one hand, the highest quantity used of the product was obtained for the tinted cream (mean = 351.70 mg/use and P90 = 521.50 mg/use). On the other hand, the lowest quantity applied was observed for the eyebrow pencil (mean = 1.48 mg/use and P90 = 2.22 mg/use).

The exposure of the panel to the tinted cream was 8.32 mg/kg bw/day. This product showed the highest exposure value among the nine investigated products, whereas the eyebrow pencil and the eye pencil had the lowest exposure values, respectively, 0.05 mg/kg bw/day and 0.06 mg/kg bw/day.

## 4 Discussion

The aim of this study was to better understand the cosmetic consumption of patients undergoing cancer treatment and to collect exposure data to five cosmetic and 10 makeup products for this specific population.

In light of the results obtained, it appears that patients undergoing cancer treatment adjust their cosmetic products consumption to their new physical conditions due to treatment's side effects. The adjustments are various; on the one hand, for some products, an increase in frequency of use is observed or even the beginning of consumption of a new product. On the other hand, for other cosmetics, frequency of use decreases, sometimes leading to a complete cessation of use.

All the observations made support the value of generating consumption data on specific populations with particular needs.

### 4.1 Skincare Product Survey

#### 4.1.1 Modification of the Consumption Habits

This study demonstrates that oncological treatments have an impact on cosmetic consumption habits. Indeed, patients undergoing cancer treatment have new needs in terms of cosmetic products due to the several side effects they have experienced. For instance, as skin dryness is one of the main side effects, patients need to hydrate more their skin. They are advised by medical professionals on the most suitable products according to the experienced side effects. Some recommendations about skin care for patients undergoing cancer treatment are available in the literature. Bensadoun et al., paper mentions some recommendations from the literature such as “educating patients on preventative measures prior to any therapy, including avoiding soaps, limiting shower time, using lukewarm water, and frequent use of emollients” [9].

#### 4.1.2 Frequencies of Use of 31 Cosmetics Products

Due to their treatment, participants have increased their frequency of use of moisturizing products such as body moisturizer, foot cream and lip balm probably in order to balance the skin dryness reported by 76.51% of the panel and the sensation of cutaneous discomfort reported by 64.43% of the participants. Indeed, as mentioned by Dreno et al., “Skin hydration relieves symptoms and reduces exacerbations of xerosis” [5]. More than half of the participants reported a hand foot syndrome following their treatment which is a common side effect after chemotherapy, widely described in literature. To reduce the impact of this side effect, medical professionals recommend applying emollients and creams as a prevention measure [10]. Therefore, skin dryness and the occurrence of the hand foot syndrome could explain the increase of the application frequency of the foot and hand creams.

As expected, some participants have stopped their use of hair products such as shampoo and hair masks during their treatment. It could be explained by the occurrence of alopecia. In comparison with data obtained from another French study [11], percentages of users are different: fewer shampoo users during cancer treatment and post‐treatment than among women over 15 years old from the general population: respectively, 79.90%, 94.60%, and 98.00%. However, mean frequencies of use of French women from the general population and participants during both studied periods (during oncological treatment and post‐treatment) are equal: 0.44 times a day.

The increase in the percentage of users of fortifying nail polish, soothing scalp lotion and mouthwash and the higher frequencies of use during treatment may be due to medical professional recommendations. Indeed, nail disorders such as nail fragility and changes in thickness can occur following oncological treatment, particularly after chemotherapy. As explained in the publication of Galimont‐Collen et al., “Application of nail polish to harden the nails can be done to prevent nail fragmentation” [12]. Moreover, medical professionals recommend using mouthwash to prevent oral mucositis which is a common side effect induced by chemotherapy, radiotherapy or targeted therapy. This side effect is characterized by an inflammation of the oral mucosa. In the paper of Antunes et al., it is mentioned that “Some authors indicate mouthwashes containing ethanol‐free chlorhexidine, due to the evidence that this should promote the mucous recovery, due to the reduction of secondary infections” [13]. Participants used more frequently the mouthwash than the general population. As a matter of fact, French women between 40 and 59 years old used the product 1.04 times a day (mean value) [14], whereas patients undergoing cancer treatment used mouthwash 1.73 times more.

Additionally, as mentioned by Kanti et al., “hair loss is often accompanied by unpleasant sensations such as pain, burning and stinging of the hair and/or the scalp”. These sensations of discomfort are the definition of trichodynia [15]. In order to lessen this sensation of discomfort, participants may have used a soothing scalp lotion. Therefore, the occurrence of trichodynia in patients undergoing oncological treatment could explain a higher consumption of soothing scalp lotion by the study participants during their treatment.

#### 4.1.3 Exposure Data: Comparison With the Literature

It was possible to compare data generated from this current work with data published in literature: exposure data published in the SCCS [7] and data from a French study: Ficheux et al. [14]. As exposure data from Ficheux et al., were available for multiple sex and age categories, the comparison was made with women of a similar age category.

The comparison is presented in Table 10.

**TABLE 10:** Comparison with data published in the literature.

Product categories		Quantity per application (Skin care products: g/use; Makeup products: mg/use)					Frequency of use (day^−1^)						Quantity per day (Skin care products: g/day; Makeup products: mg/day)				Exposure (mg/kg bw/day)			
		Current work			Ficheux et al., 2017 [14]		Current work			Ficheux et al., 2017 [14]		SCCS [7]	Current work		SCCS [7]		Current work		Ficheux et al., 2017 [14]	
		Mean	P90	P95	Mean	P95	Mean	P90	P95	Mean	P95	P90	Mean	P90	Mean	P90	Mean	P90	Mean	P90
Skin care products	Cleansing oil	5.20	10.00	—	11.00^e^	23.70^e^	1.30	2.03	—	1.07^e^	2.00^e^	1.43^a^	6.71	13.70	—	18.67^a^	1.04	2.12	1.75^f^	3.67^f^
	Face cream	0.43	0.69	—	0.89^e^	2.44^e^	1.82	2.70	—	1.05^e^	2.00^e^	2.14	0.79	1.37	—	1.54	12.06	22.18	16.01^g^	33.56^g^
	Body balm	5.27	9.05	—	10.50^c^	29.10^c^, ^e^	1.34	2.19	—	0.75^c^, ^e^	2.00^c^, ^e^	2.28^b^	7.07	13.74	—	7.82^b^	107.98	212.03	72.57^c^	166.35^c^, ^h^
	Hand cream	0.69	1.26	—	1.23^e^	2.90^e^	1.49	2.85	—	1.35^e^	5.00^e^	2.00	1.02	2.18	—	2.16	15.62	33.30	22.45^h^	57.89^h^
	Sunscreen cream	2.16	4.53	—	10.30^e^	52.00^e^	0.63	1.30	—	2.05^e^	4.00^e^	2.00	1.36	3.17	—	18.00^d^	20.68	47.22	101.32^i^	211.64^i^
Makeup products	Tinted cream	351.70	—	593.70	—	—	0.87	—	2.00	—	—	—	304.61	521.50	—	—	4.56	8.32	—	—
	Foundation	172.60	—	394.10	408.00	757.90	0.54	—	1.00	0.74	1.00	1.00	92.46	314.80	225.00	513.00	1.44	3.38	3.71	9.05
	Concealer	16.51	—	41.35	29.10	89.80	0.65	—	1.00	0.77	2.00	—	10.68	31.93	—	—	0.15	0.35	0.12	0.29
	Translucent powder	44.91	—	116.88	—	—	0.67	—	2.00	—	—	—	30.30	94.48	—	—	0.44	1.07	—	—
	Bronzing powder	32.40	—	86.18	—	—	0.81	—	2.00	—	—	—	26.36	69.14	—	—	0.42	1.02	—	—
	Eyebrow pencil	1.48	—	2.54	—	—	0.99	—	2.00	—	—	—	1.47	4.45	—	—	0.02	0.05	—	—
	Eye pencil	1.89	—	4.82	2.80	6.70	0.80	—	3.00	0.80	2.00	2.00^j^	1.52	3.70	—	5.00^j^	0.02	0.06	0.02	0.05
	Mascara	16.70	—	37.83	17.70	37.40	0.80	—	2.00	0.79	1.00	2.00	13.44	30.30	—	25.00	0.21	0.46	0.18	0.38
	Lipstick	8.04	—	21.20	8.00	16.30	1.43	—	3.00	0.88	2.00	2.00	11.46	45.45	24.61	56.53	0.19	0.41	0.10	0.25

^a^ Value corresponding to the shower gel.

^b^ Value corresponding to the body lotion.

^c^ Value corresponding to the body cream.

^d^ Theoretical value.

^e^ Value corresponding to the category of women between 40 and 59 years old.

^f^ Value corresponding to the category of women between 15 and 59 years old.

^g^ Value corresponding to the category of women over 15 years old.

^h^ Value corresponding to the category of women over 40 years old.

^i^ Value corresponding to the category of women between 40 and 70 years old.

^j^ Value corresponding to the eyeliner.

No exposure data about the cleansing oil and the body balm were available in the literature. Therefore, the cleansing oil was compared with the shower gel because they are both rinsed‐off products used on the body. The body balm data were compared with the body cream data because their texture and their application mode were similar. However, the galenic and the density of the products being different, the consumption could be slightly different.

Most quantity data generated in this current work were lower than quantity data available in the literature, except for the body balm. Indeed, the quantity applied per day by the participants were 1.76 times higher than the quantity per day published in the SCCS [7]. In the meantime, they applied the body balm with a similar frequency compared to the general population. Thus, patients undergoing cancer treatment applied more body balm at each application, maybe to reduce skin dryness. The cleansing oil, the face cream, and the hand cream were applied with a higher frequency by participants in comparison with the data published in the SCCS [7].

By comparing data from this current work with data generated from Ficheux et al. [14], it was possible to highlight that patients undergoing cancer treatment used the face cream more frequently throughout the day than women from the general population. However, the participants applied less product at each application. For the sunscreen cream, the frequency of use and the quantity applied by participants were lower than that of the general population. Medical professionals recommend to their patients to minimize their sunlight exposure during their anticancer treatment because oncological treatments, and more particularly chemotherapy, target therapy, and immunotherapy can increase the photosensitivity of patients by inducing photosensitive dermatitis [16].

#### 4.1.4 Design of the Study

Thermal cure staff were asked not to schedule body care for the participants during the consumption follow‐up in order not to bias their cosmetic consumption. However, in some cases, it was not possible. Therefore, the consumption of cleansing oil and moisturizers could be slightly underestimated.

Moreover, data from the consumption follow‐up were collected during the post‐treatment period and not during the treatment. Nevertheless, as participants were attending a thermal cure, it is possible to assume that their skin had not yet restored to its pretreatment condition and that their skin was still very dry. Therefore, consumption data generated during the study are still reliable and can be used to assess the safety of cosmetic products intended for patients undergoing cancer treatment.

### 4.2 Makeup Product Survey

#### 4.2.1 Makeup Routine Modifications

The beginning of oncological treatments affects the patient's makeup consumption. In order to take care of themselves, most of them changed their routine, sometimes due to new cosmetic needs or they modified the type of product they actually apply. A lower frequency of use was observed for 10 products out of 11 during treatment than post‐treatment. These modifications are consistent with side effects reported by the participants. They only used the eyebrow pencil more frequently during the oncological treatment to redraw their eyebrows and thus cover a side effect of their treatment that affects their face. The use of eyebrow pencil allows them to improve their self‐image, modifying, for instance. As participants had deplored lash loss due to the hair toxicity of their treatment, the mascara was applied less frequently during treatment. Some of the participants stopped or decreased their consumption of makeup products during their treatment because they were overtired, which also explains the higher frequency of use of makeup products after the end of their treatment.

#### 4.2.2 Exposure Data: Comparison With the Literature

Data collected from this current work were compared to data published in the SCCS [7] and data generated from the study Ficheux et al. [14]. The comparison is presented in Table 10.

The present study enabled the generation of exposure data for four products for which no data were available, even for the general population: the tinted cream, the translucent powder, the bronzing powder, and the eyebrow pencil.

For the foundation and the concealer, both frequency of use and quantity applied per application were lower than data from the general population. However, patients applied frequently the eye pencil more frequently than French women from the general population [14]: three times a day versus two times a day at the 95th percentile. Patients undergoing cancer treatment applied less product at each application; thus, their exposure to eye pencil is almost the same as French women's exposure: 0.06 mg/kg bw/day for patients undergoing cancer therapy versus 0.051 mg/kg bw/day for French women at the 90th percentile. The quantity of eye pencil applied per day generated from this study is lower compared to the quantity published in the SCCS [7]: 3.70 mg/day for patients undergoing oncological treatment versus 5.00 mg/day for the general population.

Participants of the study used the mascara with a higher frequency than French women from the study Ficheux et al. [14] and their exposure was higher. Moreover, the quantity of mascara applied per day by the participants was slightly higher than the data published in the SCCS [7]: respectively, 30.30 mg/day versus 25.00 mg/day. As most participants had achieved their cancer treatment, it is possible to assume that their lashes had started to grow back. The participants could have applied more product to highlight their growing eyelashes and to restore the image they had before their oncological treatment.

For the colored lipstick, both the frequency of use and the quantity per application were higher for patients undergoing cancer treatment than for French women from the study Ficheux et al. [14]. Thus, their exposure was higher: 0.41 mg/kg bw/day versus 0.25 mg/kg bw/day. However, the quantity of lipstick applied per day by the general population and published in the SCCS [7] is higher than the quantity per day obtained from the patients undergoing cancer treatment.

## 5 Conclusion

This study provided information on cosmetic product consumption habits of patients undergoing cancer treatment. It highlighted the impact of oncological treatment on cosmetic consumption. These treatments caused cutaneous side effects whose intensity can be reduced by applying cosmetic products such as moisturizing products or mouthwash.

By generating exposure data of patients undergoing cancer treatment for five cosmetic and nine makeup products, differences in quantity applied and frequency of use have been emphasized between this specific population and the general population.

As most participants were treated with surgery, chemotherapy, radiotherapy, and hormonal therapy, exposure data generated may be less representative for patients having undergone targeted therapy or immunotherapy.

The results of this study support the value of generating consumption data on specific populations with particular needs.

To complement this study, it would be interesting to conduct a new survey that will include a larger male population, as the current panel was almost exclusively female, as well as more patients under targeted therapy or immunotherapy.

An increase in the use of some cosmetic products, for which the quantities applied could not be collected in this study, has been identified. It would be useful to collect data on the quantity applied for these products, for instance: foot cream and mouthwash.

The new exposure data provided by this study could be used to conduct a more specific safety assessment for cosmetic products intended for patients undergoing oncological treatment.

## Author Contributions

**Pauline Roch‐Simon:** investigation, methodology, data curation, formal analysis, validation, writing – original draft, writing – review and editing; **Audrey Bernard:** investigation, methodology, data curation, formal analysis, validation, writing – original draft, writing – review and editing; **Sandrine Rakotomalala:** conceptualization, methodology, investigation; **Marie‐Pierre Gomez‐Berrada:** supervision, conceptualization, methodology, review and editing; **Pierre‐Jacques Ferret:** supervision, review and editing.

## Ethics Statement

The authors confirm that the ethical policies of the journal, as noted on the journal's author guidelines page, have been adhered to. This study is a noninterventional, observational, real‐life study in which participants used already commercialized cosmetic products according to their everyday habits. In accordance with the Regulation N°1223/2009 of the European Parliament and of the Council of 30 November 2009 on cosmetic products (Chapiter II—Article 3) [6], the safety of these products has been assessed prior to their marketing. In view of the study design and the above considerations, an ethics committee is not necessary. Before their inclusion in the study, all participants were given an information document including a description of their rights about the processing of their personal data, in accordance with the Regulation (EU) N°2016/679 of the European Parliament and of the Council of 27 April 2016 [17] on the protection of natural persons. Each member of the panel provided signed consent prior to the beginning of the study.

## Conflicts of Interest

The authors declare no conflicts of interest.

## Data Availability

The data that support the findings of this study are available on request from the corresponding author. The data are not publicly available due to privacy or ethical restrictions.
